# The Influence of Climate and Other Agents on the Human Constitution, with Reference to the Causes and Prevention of Disease among Seamen

**Published:** 1844-07-01

**Authors:** 


### The Influence of Climate, and other Agents, on the Human Constitution, with reference to the Causes and Prevention of Disease, among Seamen. By *Robert Armstrong*, M.D. F.L.S. Deputy Inspector of Hospitals. Pp. 207. London, 1843.

The substance of the present work was originally prepared by the author in the form of lectures intended to be delivered by him, when in charge of the Naval Hospital at Jamaica, to the various medical officers serving upon that station. He therefore directed attention principally to those subjects likely to be useful to the younger branches of the profession upon their first entering the service, often fresh from the medical schools, thoroughly imbued with the various theories there prevailing, but little if at all acquainted with actual practice. He much wished to caution them against falling into mere careless routine, accepting certain vague terms as sufficient explanations of the phenomena passing around them, to the neglect of the employment of their own powers of observation and thought. Just as he was upon the point of delivering them, however, his appointment to the Naval Hospital at Plymouth, threw him into a different sphere of action, and his notes were put aside. The manuscript having been perused with advantage by several surgeons of ships ordered to the West Indies, Dr. Armstrong has, at their invitation, revised it, and adding some further illustrations, published it in the present form. The first nine chapters relate to various matters connected with the production of disease among seamen, and the hygienic precautions adapted to warding it off; the three last treat of fever in general, and of the yellow fever of Jamaica in particular.

We proceed to extract such portions as we think will interest our readers.

Speaking of the effects of locality and mode of life in inducing varieties in animals, and the human race, the author observes:—

"The influence of warm climates is apparent after a few years residence within the tropics: Europeans lose their sanguineous complexions, and acquire the power of resisting the heat better than the new comer. This power of accommodation to circumstances, arises from a corresponding change in the functions of life; and which is usually attributed to the individual having undergone the process of seasoning;—a process of which the most vague opinions seem to be entertained. Even within the limited extent of our own country, we observe the influence of local situation, on comparing the natives of mountainous districts with those in the low country. Among the former, examples of tall individuals are less frequently met with. In the mountaineers, the chest will be found more expanded, and the circulation more vigorous; the muscular system more developed, and the forms harsher. The bilious temperament prevails; and they possess a greater quickness of temper and energy of character.

"This greater development of the respiratory apparatus, seems to result from the physical necessity of situation. In the habit of ascending and descending heights of considerable elevation, and generally breathing a more attenuated atmosphere, the organic apparatus acquires greater capacity and vigour, to enable it to afford the necessary supply of atmospherical oxygen. In level countries, on the other hand, the inhabitants are usually of taller stature: the general contour of the figure is more rounded, from the preponderance of the cellular and adipose tissues. The prevailing temperament is the sanguineous. The chest, when viewed in relation to the size of the body, is less expanded; and they are not so well adapted to undergo fatigue when removed from their native districts. Differences of situation even impress upon the constitution a disposition to particular diseases. Scrofula and consumption, are of more frequent occurrence in the plains and agricultural districts, than in the mountainous parts of the country.

"Let us observe man under other circumstances, as in the crowded districts of a great city, or assembled in masses in our manufacturing towns. Here he is employed in occupations in which dexterity rather than strength, is required; and breathing an impure atmosphere with bad lodging and bad food, the body does not attain its full development, and he is deficient in muscular power. This acquired constitution of body is transmitted to the offspring. His diseases are also modified by his condition; and the constitution has not the same power of reparation in cases of injury or disease. This permanent deterioration in the qualities of the race, has been clearly proved by their disqualifications for those offices, where a certain standard of size and strength is required. It is asserted that men from Spitalfields, and other crowded districts of London, who come forward as candidates for employment in the police force, fall short of the required standard; and that two out of three are rejected, as physically unfit for service.

"'It is observed in some of the worst conditioned of the town districts, that the positive number of the natives of the aboriginal stock continually diminishes, and that the vacancy, as well as the increase, is made up by emigration from healthier districts. In a late enumeration of the settled inhabitants of Westminster, it appeared that not more than one third of them were natives of London. Sir James Mc Grigor, the Director General of the Army Board, stated the fact that a corps levied from the agricultural districts in Wales, or the Northern counties of England, will last longer than one recruited from the manufacturing towns, from Birmingham, Manchester, or near the metropolis. Indeed, so great and permanent is the deterioration, that out of 613 men enlisted, almost all of whom came from Birmingham, and five other neighbouring towns, only 238 were approved for service.'* "

Dr. Armstrong observes, that although the animal economy is enabled to regulate its supply of animal heat according to the temperature of the climate or the season, yet some time is requisite to accustom it to any considerable change, and the period of such transition is one of susceptibility to disease. When a large ship of war is commissioned, men are received from all quarters, and comprise not only good and experienced seamen, when they are to be obtained, but also volunteers of every possible variety of constitution, who have frequently been living in a dissipated, profligate, or destitute manner. While the equipment is going on, the working parties in the dock-yard are much exposed to the weather, and catarrhs, febrile and rheumatic attacks, &c. prevail among them; but, as the diet is good and abundant, the ship's company becomes much improved in appearance; while some of them, from the contrast to their former fare, become diseased through repletion. The marines enjoy invariably a higher state of health than the seamen, owing to their restriction to their barracks when on shore, and to their strict order and regularity. When the ship pro-

* Poor Law Commissioners Report, 1842.

ceeds to sea, a complete change in the habits of her company ensues. Their diet is salt meat, and their rest disturbed by the alternation of watches; and if stormy weather occurs inflammatory affections soon manifest themselves. If the vessel be destined to the West Indies, a tropical temperature, involving a change of 30° or 40° Fahr., if she sailed in the winter, may be attained in a fortnight.

"On approaching the warmer latitudes, the looks of sailors improve; as the ship glides along before the North-east trade-wind, under a clear and blue tropical sky, days often pass without a sail requiring to be shifted. The temper of the ship's company becomes more cheerful—a greater degree of alacrity is observed in their movements—the skin becomes softer, elastic, and more vascular, with an increase of perspiration,—and they enjoy a superabundance of health, if the phrase may be allowed. In some cases of indisposition, so slight as scarcely to deserve notice, a few ounces of blood have frequently been drawn as an experiment. It exhibited on every occasion, the buffy coat, as we find in other cases of excitement. The crassamentum was firm, and evidently contained a larger proportion of fibrine than usual."

Under these circumstances, the amount and quality of diet allowed continuing the same, much evil would probably result but that Nature, more wise than man, diminishes the assimilative powers. As it is, however, some derangement of the secretions, accompanied by constipation, usually occurs: and when disease does appear, it does so usually in the form of inflammatory fever with determination to the head. Boils and prickly heat exert sometimes a useful derivative influence.

"Supposing the East Indies to be the destination of the ship, they pursue their course to the southward, and stretch towards the South American continent. On getting beyond the limits of the South-east trade, they steer to the South-east, and run into the latitude of 36° or 38° South, where strong westerly winds are found to prevail. In fourteen days, or three weeks, they are again wafted into a cold climate, where the temperature is from 36° to 40° Fahr. and sometimes at the freezing point. The ship continues on this parallel, across the Southern Ocean, for a distance of three or four thousand miles. During this run, nothing is seen to divert the mind; and the countenances of the persons embarked will be found to have undergone a change, and assumed a certain haggard expression. If examined closely, the gums will be seen to have lost their natural red hue, appear shrivelled, and present a whiteness around the roots of the teeth.

"The diseases which now appear, especially if the weather prove wet and cold, are pneumonia, rheumatism, catarrh, and diarrhœa: the accompanying fever being of the low, nervous type. In some cases of slight indisposition, a few ounces of blood were drawn from the arm, rather by way of experiment than from necessity; and it was found to be less florid, and slower in coagulating, than within the tropics; the crassamentum was easily broken down between the fingers, and evidently contained a smaller proportion of fibrine or coagulable lymph. This was observed in seven convicts under 30 years of age, and three soldiers, out of a party of 40, embarked as a guard. The former, although prisoners, were allowed to come on deck before the mainmast when they pleased, and occasionally assisted in working the ship of their own accord. The soldiers did duty as a guard, and were on full rations; while the convicts were on two-thirds, or six men had the allowance of four seamen, but they had many comforts in addition.

"If India be the place of destination, on reaching the 70th or 80th degree East Longitude, they direct their course North, and soon get to the South-east trade-wind, which carries them into the Bay of Bengal. Thus, in the short space of three or four months, the ship's company have experienced two Summers and two Winters.

"Although actual disease may not have appeared during these rapid and extreme changes of climate, it cannot be doubted, that a certain excitability of constitution is generated, which renders the body more susceptible of impression from the ordinary exciting causes of disease." 32.

The author is no believer in the existence and agency of *malaria* in the production of disease. Its production by the miasmata generated in marshy and other unhealthy districts and places, such as ships' holds, &c., he considers as quite unproved. On two occasions, when fever was prevailing extensively in Batavia, he exposed himself at night purposely to the exhalations proceeding from localities possessing all the physical characteristics said to be favorable to the production of malaria, with impunity. He concludes this part of the subject as follows:—

"In the preceding detail, it has been shewn, that we are utterly ignorant of this animo-vegetal poison; that no two authors agree respecting its nature, the circumstances under which it is generated, or its effects upon the human body. How then are we to reconcile these discrepancies with the existence of a material said to be possessed of such tremendous activity—a material of whose existence we have no proof, and of whose physical qualities we have no knowledge? What grounds have we for supposing, that the materials, which are said to give rise to this mysterious and invisible something, should continue for indefinite periods of time in a state of inactivity, as far as regards its production, and yet be undergoing the changes inherent to organized matter all the while? How can such materials let loose for a season exhalations which kindle up the most untractable diseases, and again become dormant? Such capricious movements among the particles of matter are at variance with the established laws which regulate chemical action in all other cases.

"All our reasonings, therefore, respecting the nature of these miasmata, are founded on an assumption; and consequently want that certainty which constitutes the only sure ground of belief. The existence of this material can only be regarded as an assertion—a species of evidence which is not admissible in the inductive sciences; and until proofs be brought forwards of a very different kind from any yet adduced, we cannot acknowledge, without admitting contradictions and impossibilities, the existence of this vegeto-animal poison. The belief in such a material has an injurious effect, because it tends to suppress all observation and inquiry into the various causes of disease; whereas, by close observation, and the correct application of well-known principles, we shall soon find, that there are many powerful causes operating upon the bodies of men in warm climates; and which are fully sufficient to account for the ravages of disease, without the necessity of calling in the aid of aereal and unsubstantial phantoms." 71.

Among the powerful causes alluded to in the above extract, Dr. Armstrong includes the various meteorological changes in the state of the atmosphere, as evidenced by the varying condition of its electricity, magnetism, and weight. Then again the "sol-lunar influence" should by no means be disregarded. He remarks further:—

"These investigations appear to have been entirely neglected or overlooked by medical officers in the public service, yet there is no class of men who have greater opportunities of contributing to scientific knowledge. The state of the atmosphere and the intimate connexion which subsists between it, and the condition of the various functions of life, are of the utmost interest and importance, both in health and disease; and in this extensive, and yet untrodden field, a rich harvest may be reaped. An account of the temperature of the air night and morning, as inserted in the journal, without reference to other circumstances, is of comparatively little value, and cannot afford data for any practical results. The dryness and humidity, the sudden changes of temperature, and of the electrical condition, should be carefully observed. The radiation of heat in warm climates; the geological structure of the country; and the periods of the accession of diseases, are also objects of the greatest interest, and by common industry and observation, much valuable information may be obtained."

Although we cannot agree with the author in his views respecting the non-existence of malaria, we believe great good must result from directing the attention of medical officers to the various other potent causes of disease, many of which are susceptible of mitigation or removal. Thus the change of habits, diet, &c., on arrival at the ship's destination, the laborious employments then performed aboard her, under exposure to the sun's rays, together with indulgence in spirituous liquors, give rise to much disease. Not only is exposure to a tropical sun a fertile source of mischief, but the variation of temperature is so likewise. Thus the ship's company, while in harbour, is crowded into so small a space that each man has seldom more than 16 inches at his disposal. The temperature of the lower deck has been often observed at 90° or higher, while the air on deck did not exceed 70° or 75°, with a strong breeze loaded with moisture blowing. On coming on deck a slight shiver or chill occurs. "Getting out of bed in a state of profuse perspiration, they will remain in this cold air for a time to cool themselves. On returning to their hammocks, they feel chilled and restless, and some febrile attack succeeds. Many cases of fever, acute rheumatism, pneumonia, and dysentery could be clearly traced to this cause." The great increase of sickness during long-continued hot weather has been frequently remarked; and the statistical report shews this to be the case, not only in the West Indies, but in the East, the Mediterranean stations, &c. Even moderate exposure to the sun, when conjoined with active employment, is highly injurious.

The author considers the changes of temperature, to which the soldier and sailor are exposed in hot climates, have not been sufficiently dwelt upon.

"These changes are greater, it is true, in some months than in others, but they occasionally occur at all seasons. At Port Royal, I have repeatedly observed a reduction of from 15° to 20°, in the space of half an hour. About sun-set, when the sea-breeze had died away, and the air was perfectly calm, a thermometer placed in an open balcony would indicate 86° or 90°. A strong land breeze charged with moisture, would now come down in strong gusts from the mountains, and reduce the thermometer to 68° or 70°, and sometimes lower. At midnight, and at sunrise, in the morning, the thermometer has often been observed at 70° and 72°: the air was then cool and pleasant, and half an hour's walk along the beach was most invigorating. Between nine and ten o'clock, when the land-wind had subsided, and before the sea-breeze had set in, the thermometer would be from 84° to 90.°

"The duck frocks and trowsers afford a sufficient covering during the day, when the temperature in the shade is from 80° to 86°, and many degrees higher in the sun. In the evening, when the temperature is reduced by the cold land-wind saturated with moisture, and consequently its conducting power greatly increased, warmer clothing is absolutely necessary. During calm, clear nights, the radiation of heat, and evaporation from the surface of the body, farther co-operate as cooling processes." 87.

These vicissitudes, the author considers, explain the occurrence of many fevers acquired during night-duty, and attributed to miasmata. Parties, so employed, when supplied with spirits or other stimuli, do not suffer in the same way upon exposure to night-air.

Moral causes have ever had a great effect in inducing or warding off disease in naval and military expeditions. Confidence in the commander and in the success of the undertaking, exerts a powerfully conservative effect upon the health, even in the midst of privations, while active operations have a no less beneficial tendency. Where, however, the officers show indecision in their proceedings, success does not follow the efforts made to achieve it, or a long period of inaction after continued successes is unavoidable, then the cases of disease become very numerous. The bad reputation for salubrity which is attached to the Coast of Africa induces a dread among seamen and officers, which powerfully facilitates the inroads of disease. In the West Indies, too, it has long been observed that those who entertain the greatest dread of the climate, first fall victims to fever, while those who take common prudential precautions merely, usually escape. Dr. Armstrong instances also the late Niger Expedition as a strong example of the ill effects resulting from the combined operation of a depressed condition of the moral powers, (engendered, in a great degree, by over-precaution,*) fatigue, and exposure.

" In this ill-fated expedition, various circumstances conspired to excite gloomy

---

* We extract a passage from a well-written review of M'William's History of the Niger Expedition, contained in the pages of our able contemporary, the Athenæum, (1843, p. 603.)

" This is the history of the mortality attending an expedition, in which the greatest pains seem to have been taken to keep the minds of all engaged in it intent on disease and death. Precautions were carried to such an extent that even the very air was physicked. Dr. Reid's system of ventilation—a fine example of science for the million—was applied to the vessels built for the expedition ; but as it seemed that ventilating with unwholesome air, was only beating up with much ado from Scylla to Charybdis, an empirical method was hit upon of freeing the air from the impurities with which Nature's chemistry is supposed to have filled it. Accordingly, the air gathered in the wind-sails aloft was conducted into a chamber where its floating poisons were extinguished, or supposed to be extinguished, by the superior poison of chlorine ; and the zephyrs thus sweetened, *secumdum artem*, with the balmy influence of quick-lime and sulphuric acid, were diffused below decks.

" Thus the men under cover respired only medicated air—on deck, of course, they were obliged to breathe fresh air unmedicated, but in that case they were taught the danger of inhaling air which the Doctor had not overhauled. 'Those who are obliged to be on deck on duty (we quote Capt. Trotter's general orders) will be supplied, when in unhealthy localities, with respirators.' Supplied with respirators ! Alas ! poor Jack ! how wretched and chapfallen you must have felt, when obliged to breathe through a respirator ! The quid shut out, the yarns shut in, your heart and soul kept under gratings—why you must have thought it hardly worth while to breathe at all, when, with a chest capable of respiring great guns, you were compelled to discharge it through contemptible scuttle-holes."

anticipations. The professed object was the introduction of civilization into Africa, and which, no doubt, originated from the most philanthropic motives. Iron steamers were built for the special service, and the officers were appointed from their experience and local knowledge of the country, or interest in the cause. The seamen were volunteers, and received double pay. Stores of all descriptions were most liberally supplied; and, indeed, in this respect, there appeared to be no restriction. Wine and porter were put on board by hundreds of dozens, and large supplies of preserved meats and soups. A ventilating apparatus was fitted to propel air into every part of the vessels; and to purify that air, materials for the production of chlorine were provided by hundred weights.

"On minutely examining two of these vessels, when on the eve of departure from Plymouth, the accommodations of the officers and men appeared small, and were too much crowded. There was no berth for the sick, and they were completely filled with stores. The men appeared to be in good spirits, but it was evident, that they considered the enterprize hazardous; and, indeed, the whole tenor of the conversation of the officers showed a perfect conviction in their minds, that sickness was expected as a matter of course. The unusual preparations that had been made constantly reminded the seaman, that he was entering upon a service different from all others; and in a climate, he was led to believe, pregnant with pestilence and death. This gave rise to gloomy forebodings, and these feelings were still farther heightened, by the frequency of religious exercises on board the ships, and exhortations to prepare for another world. This was unprecedented in the naval service. The seaman took no interest in the civilization of the negro, and was out of his element. If placed alongside the enemy, he would understand his position, and be able to clear his way; but here he was subjected to a species of mental torture, but too well calculated to undermine both his moral and physical energies."

After describing the course of the vessels, and noticing the atmospheric vicissitudes, and the fatiguing occupations alternated with periods of inaction of their crews, the author continues :—

"The causes which had been in operation for a period of three months, (by which time they had entered the Niger,) had now produced their effects, and rendered the system more susceptible of impression from any new cause, whether of a physical or moral nature. Their hopes during the voyage had been raised to the highest pitch, but they now saw but a faint prospect of their sanguine anticipations being realized. Fever appeared, and spread rapidly through the ships. The tolling of bells to prayers and funerals, amid the general gloom, was not calculated to inspire the mind with confidence and hope; and, according to the testimony of an officer present, a feeling of despondency and dismay prevailed throughout the ships. From the number of sick, the prosecution of the enterprize was abandoned, after the loss of many valuable lives.

"It is unnecessary to adduce farther examples. Those already mentioned are sufficient to show, that moral causes exert a powerful influence over the health of large bodies of men, as well as of individuals; and that promptness and decision in the leader, the maintenance of cheerfulness of mind, and confidence in their own powers, are the most essential elements of success in naval and military operations." 98.

The ease with which what is called *seasoning* in hot climates is accomplished, much depends upon the habits of life pursued. Thus, temperance in eating and drinking is highly important. Men who have passed the meridian of life in England suffer less from heat than younger persons; and indeed life might often be prolonged by removal from a cold to a warmer climate, at the period when the power of producing animal heat becomes more feeble. Sir Joseph Banks, for years before his death, afflicted with paralysis of the lower limbs, lived in a temperature of 80°. Changes of climate produce however much effect upon the constitution beyond that which is immediately visible. Thus men of sound constitutions may resist every possible vicissitude with success, as far as the positive production of disease is concerned, and nevertheless the "wear and tear" they have been subjected to is visible in the premature old age induced. From this cause few seamen continue fit for active service after they have attained the age of forty-five, when indeed they appear ten or fifteen years older than they are. So, too, the mortality of the troops in the West Indies increases with the advance of age, because, owing to the changes and irregularities they have been subjected to throughout their career, they possess less and less power of resistance to injurious impressions. Climate, however, is not the sole cause of mortality; for the black troops in the West Indies, natives of the country, furnish the highest ratio of mortality of any others, and three times as great as that of the native troops in the East Indies. Dr. Armstrong attributes this to the full diet of animal food which is furnished them in the West Indies, quite unsuited to their former and natural habits, and which the religious habits of the East Indian troops forbid their indulgence in. And this is a grave error in the management of our own seamen, who are allowed in a tropical region the same diet that is found equal to the wants of their systems in a cold climate. The quantity of meat should be diminished, and either vegetables, if necessary, or an equivalent in money, substituted. Much advantage has resulted by giving tea in the afternoon in lieu of a portion of the spirits. The indulgence in stimulating meats and drinks so prevalent among officers is a fertile source of disease. Spirits are, however, very useful when the men are subjected to much boat service by night, sleeping on shore exposed to a moist atmosphere, &c. Tobacco, too, on such occasions, acts as a grateful and gentle stimulant.

Dr. Armstrong thinks little utility arises from fumigating ships with chlorine, &c. and believes the noxious gases, against which this is directed, to reside chiefly in the imagination. He thinks bad smells, &c. can arise only from the neglect of cleanliness; but this must be a rare occurrence, as the opposite extreme of excessive cleansing and rubbing is usually pursued. Plenty of water and a good ventilation of the decks are all he considers necessary. Dr. Reid's apparatus is too bulky for small, and cannot be required for large ships. The author suggests a simpler plan by supporting the combustion in the ship's coppers with air drawn from the hold.

Some reform is clearly called for in reference to the *change of clothing* of seamen. At certain seasons orders arrive for the substitution of duck for cloth trousers and the reverse, and this irrespective of the actual temperature which may be prevailing. In tropical countries the light clothes worn during the employments of the day with comfort and advantage become quite improper in the evening; and whenever cloth or flannel have been used instead great advantages have resulted. Flannel and blankets are especially necessary, where night service is frequent. So, too, when the ship is ordered home to be paid off, a liberal supply of warm clothing should be issued to the men when she reaches lat. 30° N., to enable them to meet the cold which awaits them.

In bad weather seamen suffer grievously from *damp clothing*, having nowhere to dry it, it is put on again nearly as wet as when taken off. Not only would the seamen's health be improved by due attention to affording him facility for drying, but the clothes, which are now thrust away wet in bags to become mouldy and injured, would be much better preserved.

*Personal cleanliness* is often too much neglected by the ship's company, most contenting themselves with washing the face and neck. When the weather allows, bathing should be freely encouraged; and the danger which attaches to one or two persons bathing in warm latitudes, on account of the presence of sharks, does not exist when a great number partake of the amusement, even in the very haunts of these animals, the splashing and commotion of the water seeming to alarm them. The decks should not be kept constantly wet or damp. Washing them once a week, and drying them rapidly with hot sand, or a swing stove, answers every purpose of cleanliness in cold or damp climates. In warm dry weather this may be performed oftener. Dry-rubbing with sand and stones effectually cleanses.

*Yellow Fever of Jamaica.*—Passing over the author's remarks upon fever in general, which contain little novelty, we extract a few observations upon the epidemics of Jamaica which he witnessed.

These epidemics, he conceives, were not produced by causes connected with the soil or locality, but were dependent upon adventitious circumstances and individual susceptibility. One ship would remain free of fever, although in communication with another at the same anchorage in which it was raging. The attack was often induced by active exercise and exposure to the sun or night air. When the fever had become developed great determination to the head and oppression of the respiratory organs occurred. About the third day, these, with the heat of skin, become diminished, and on the fourth day, the conjunctivæ and surface of the body, to a greater or less extent, acquire the yellow colour which has furnished the disease with its pre-name. About the fourth or fifth day pain sometimes subsides entirely, the pulse does not exceed 70 or 80, while the temperature of the body is natural and the skin and tongue moist. The patient, if not prevented, arises and dresses himself. "A medical man, unacquainted with the insidious nature of this disease, would immediately pronounce the patient convalescent, and more than one surgeon, on his first visit to the hospital after arrival from England, expressed the greatest surprise and incredulity, on being informed that the patient would not survive 24 hours." This "fatal lull," as it is termed, continues for three or more days sometimes. Eventually, delirium, variable pulse, reduction of the natural temperature, and other marks of prostration, occur. The *black vomit* seldom appears before the third day, or when the violence of the original febrile symptoms has subsided. It is a singular fact, too, that the dark matter forming the black vomit is found in the stomachs of those who have died of other diseases than fever.

From very numerous post-mortem examinations the author concludes—

"That the yellow fever of Jamaica is a disease of the whole system, in which every organ is more or less affected; with local determinations of blood to particular parts, succeeded by congestion of the vessels; that the tumultuous actions of the nervous and vascular systems exhaust the vital energies, arrest or suspend the secretions, and but too often terminate in the death of the patient." 181.

*Treatment.*—The greatest diversity of opinion prevails with respect to this. According to Dr. Armstrong's experience, if the patient is subjected to active treatment sufficiently early he may be frequently saved ; but that after the second or third day, the opportunity for putting this into force has passed away, and all measures are too often useless. Free and quickly repeated bleeding should at once be had recourse to, and although from 20 to 40 oz. will usually suffice, other cases may require the loss of 90 oz. The patient should be immersed in a warm bath, and the skin thoroughly cleansed by soap and flannel. Next administer an active purge containing several grains of calomel. In many cases rapid recovery results. Where, however, the symptoms are only partially relieved, leeches or ice to the head, and stimulation to the skin by a warm salt-water bath, or ammoniated liniments, should be had recourse to, giving laxatives from time to time.

In 42 cases, tartar emetic, combined with calomel, was used with evident advantage, preceded by bleeding and followed by purges. It was never given later than the first 24 hours, and prevented the necessity of excessive abstraction of blood. Difficulty arises in affecting the gums with mercury, when given from the commencement of the disease, but they become more easily affected when antimony is also given. It is very doubtful whether the mercurial treatment has any superiority over any other. Calomel combined with opium was found useful in torpid conditions of the nervous and sanguiferous systems, an occasional turpentine purgative being also given. There is no truth in the supposition that ptyalism affords any protection, for syphilitic patients, while salivated, become affected with the fever.

In the advanced stages of the disease, whatever is done, death still usually occurs. Tonics and stimuli are to be given ; and, indeed, the great object is to maintain the heat of the body by internal stimuli and external warmth. A pint of brandy, and a bottle or two of porter, and even larger quantities may be consumed daily without injuriously augmenting the pulse or heat of surface.

It is an error to suppose that having had the fever once always gives an immunity against a second attack. It does so usually, but not always. Dr. Armstrong says :—

> " It must be admitted, however, that men who have already had an attack of fever, are much less liable to suffer from it again than new comers. If, however, they returned to England, and joined another ship ordered to the West Indies, they appeared as liable to suffer as those who had never before been on the station."

Although not prepared to deny that the disease may never become *contagious,* the author is clearly of opinion, that it is not so under the circumstances in which it is usually observed.

This book has no pretension to be considered a complete treatise upon the subjects it treats of. It contains, however, some good practical remarks, and will prove useful to young naval surgeons.

